# The Anthropocene, global change and sleeping giants: where on Earth are we going?

**DOI:** 10.1186/1750-0680-1-3

**Published:** 2006-06-27

**Authors:** Will Steffen

**Affiliations:** 1 Director, Centre for Resource and Environmental Studies, The Australian National University, Canberra, Australia

The "climate problem" has come to the fore in public policy debates over the last year or so. The continuing high temperatures, the spate of intense tropical cyclones and deepening droughts in some parts of the world have focused attention on the issue of defining "dangerous climate change" [1]. This is often conceptualised as an upper limit to the rise in global mean temperature, for example, 2°C above pre-industrial levels, which in turn leads to a back calculation of the permissible concentration of CO_2 _in the atmosphere and then to the trajectories of the corresponding maximum anthropogenic carbon emissions.

Although a very important exercise, this approach to defining dangerous climate change can itself be dangerous, in particular because it often ignores the systemic nature of the global environment. Feedbacks and nonlinearities are the rule, not the exception, in the functioning of the Earth System [2], and in this Anthropocene era, where human activities have become a global geophysical force in their own right, there is no doubt that surprises await those who apply linear logic to the climate problem. The carbon cycle is centrally involved in many of these feedbacks and nonlinearities.

Here we briefly review several of the more important so-called "sleeping giants" in the carbon cycle, processes that have the potential to accelerate the rate of warming beyond that attributed to human emissions of greenhouse gases [3]. The first of these is based on the impact on soil respiration of rising temperature and changing soil moisture, an example of a response of ecosystem physiology to climate change. Although there is still debate about the magnitude of the increase in soil respiration with temperature, and whether there are compensating effects of enhanced plant growth due to mobilisation of nitrogen in the process, the general consensus is that increasing temperature will cause an increase in the emission of CO_2 _from soil carbon [4].

A second "sleeping giant" is the increase in disturbance in terrestrial ecosystems, often associated with pulses of carbon to the atmosphere. The most notable of these are wildfires and pest outbreaks, both sensitive to both warming and changes in the moisture regime. Although these are natural phenomena in the dynamics of terrestrial ecosystems, an increase in the frequency or extent of these disturbances results in a net loss of carbon to the atmosphere. Observations of the large areas of boreal forest in the northern high latitudes suggest that over the past couple of decades, these forests have experienced enhanced rates and/or areas of disturbance, and hence their capacity to act as sinks for atmospheric CO_2 _has weakened significantly [5]. Human-driven land-use and land-cover change is another type of disturbance with important implications for the terrestrial carbon cycle, with perhaps even more complex dynamics than wildfires or pest outbreaks.

Coupling global climate models to carbon cycle models that account for these responses to a warming climate provides first estimates of the magnitude of these carbon cycle feedbacks [6]. The results vary considerably, from an additional 20 ppmV CO_2 _in the atmosphere by 2100 to over 200 additional ppmV. However, all simulations showed a positive feedback – the warming is accelerated beyond that due solely to anthropogenic carbon emissions. In a worst case scenario, the additional warming by 2100 due to these carbon cycle feedbacks could be about 1.5°C.

Potentially important carbon cycle feedbacks do not, however, end with soil respiration, fire and insect outbreaks. Permafrost soils in the high latitudes of the northern hemisphere and moist peatlands in both the tropics and the high latitudes contain hundreds of gigatons of carbon that is currently stored away from contact with the atmosphere. However, both of these pools of carbon are vulnerable to rising temperatures, which could melt much of the current permafrost areas and dry out peatlands, leading to the emission of CO_2 _and CH_4 _to the atmosphere [7].

Carbon in its elemental form – soot or black carbon – plays a complex role in the climate system. For example, black carbon produced by wildfires and stored in soils or water systems acts as a sink for carbon; however, fine soot particles released to the atmosphere can also act to warm the climate further, unlike many other aerosol particles.

The marine carbon cycle may also provide surprises in the future. The dissolution of atmospheric CO_2 _in the surface waters of the ocean increases their acidity through the formation of carbonic acid. This, in turn, affects the saturation state of calcium carbonate, a basic building block for organisms such as corals, shellfish, sea urchins, starfish and some forms of phytoplankton that form calcium carbonate shells [8]. The rising acidity of the ocean will no doubt have significant effects of the trophic structure of marine ecosystems, but will also affect the functioning of these systems. The implications for the marine carbon cycle are not yet clear, but a weakening oceanic sink for carbon due to the increasing concentration of carbonic acid is a likely result.

In an Earth System context the carbon cycle can act as a buffer to keep the planetary environment within well-defined limits, but if critical thresholds are crossed, the carbon cycle can act as a giant flywheel that helps to push the Earth System into another state. As the 21^st ^century progresses, the concentration of CO_2 _in the atmosphere will depend not only on the amount of anthropogenic emissions, but increasingly on the response of natural carbon cycle dynamics to the changing climate. Clearly there is an urgent need for the carbon cycle policy and management communities to move beyond the simple human emissions-atmospheric CO_2 _equation and to take a much more holistic view of the carbon cycle – its natural dynamics, feedbacks, nonlinearities and potential surprises.
